# Prognostic value of beta-blocker doses in patients with ventricular tachyarrhythmias

**DOI:** 10.1007/s00380-021-02018-3

**Published:** 2022-01-24

**Authors:** Tobias Schupp, Sevil Ziyadova, Julius Reinhardt, Yusuf Ugur Sag, Max von Zworowsky, Linda Reiser, Mohammad Abumayyaleh, Kathrin Weidner, Ahmad Saleh, Kambis Mashayekhi, Thomas Bertsch, Mohammed L. Abba, Ibrahim Akin, Michael Behnes

**Affiliations:** 1 Department of Cardiology, Angiology, Haemostaseology and Medical Intensive Care, Faculty of Medicine Mannheim, University Medical Centre Mannheim, Heidelberg University, Mannheim, Germany; 2 European Center for AngioScience (ECAS) and German Center for Cardiovascular Research (DZHK) Partner Site Heidelberg/Mannheim, Mannheim, Germany; 3 Department of Cardiology and Angiology II, University Heart Center Freiburg, Bad Krozingen, Germany; 4 Laboratory Medicine and Transfusion Medicine, Institute of Clinical Chemistry, Nuremberg General Hospital, Paracelsus Medical University, Nuremberg, Germany; 5 Third Department of Medicine, University Medical Centre Mannheim (UMM), Faculty of Medicine Mannheim, University of Heidelberg, Mannheim, Germany

**Keywords:** Ventricular tachycardia, Ventricular fibrillation, Mortality, Beta-blockers, Target dose, Medical treatment, Pharmacological drugs

## Abstract

The study investigates the prognostic significance of beta-blocker (BB) dose in patients with ventricular tachyarrhythmias. Limited data regarding the prognostic impact of BB dose in ventricular tachyarrhythmias is available. A large retrospective registry was used including consecutive patients on BB treatment with episodes of ventricular tachycardia (VT) or fibrillation (VF) from 2002 to 2015. Discharge BB doses were grouped as > 0–12.5%, > 12.5–25%, > 25–50%, and > 50% according to doses used in randomized trials. The primary endpoint was all-cause mortality at three years. Secondary endpoints comprised of a composite arrhythmic endpoint (i.e., recurrences of ventricular tachyarrhythmias and appropriate ICD therapies) and cardiac rehospitalization. Kaplan–Meier survival curves and multivariable Cox regression analyses were applied for statistics. A total of 1313 patients with BB were included; most patients were discharged with > 25–50% of BB target dose (59%). At three years, > 12.5–25% of BB target dose was associated with improved long-term mortality as compared to the > 0–12.5% group (HR = 0.489; 95% CI 0.297–0.806; *p* = 0.005), whereas higher BB doses did not improve survival (> 25–50%: HR = 0.849; *p* = 0.434; > 50%: HR = 0.735; *p* = 0.285). In contrast, the composite endpoint and risk of rehospitalization were not affected by BB target dose. In conclusion, > 12.5–25% of BB target dose is associated with best long-term survival among patients with ventricular tachyarrhythmias. In contrast, risk of the composite arrhythmic endpoint and risk of cardiac rehospitalization were not affected by BB dose.

## Introduction

Beta-blockers (BB) were demonstrated to decrease all-cause mortality and risk of sudden cardiac death (SCD) in various randomized controlled trials (RCT) including patients with systolic heart failure (HF), acute myocardial infarction (AMI) and arterial hypertension [1–3]. These studies commonly investigate the prognostic impact of BB therapy for primary prevention of SCD [4]. In contrast, less data is available regarding BB treatment for secondary prevention of SCD. Using a large registry including patients with ventricular tachyarrhythmias, we recently demonstrated that BB therapy improves survival secondary to ventricular tachyarrhythmias [5]. Thus, the prognostic impact of BB therapy may be dose-dependent. The effect of different BB doses was, however, beyond the scope of RCT in the field of BB therapy. However, during routine clinical care, BB doses are commonly lower than doses used in RCT and up-titration of BB therapy was reported to occur infrequently [6, 7]. The dose-dependent impact of BB therapy was already investigated within registries including HF and AMI patients [8, 9]. Prognosis in patients treated with different BB doses based on target doses used in RCT was recently investigated in a multi-center registry of almost 7000 AMI patients [10]. At two years of follow-up, higher BB doses were not associated with improved all-cause mortality as compared to low daily BB doses, whereas survival was improved in patients with BB therapy as compared to those without.

To the best of our knowledge, the prognostic role of BB target doses for secondary prevention of SCD was not yet investigated, even not in patients presenting with ventricular tachyarrhythmias. Therefore, the present study evaluates the prognosis of patients with ventricular tachyarrhythmias treated with > 0–12.5%, > 12.5–25%, > 25–50%, and > 50% of BB target dose according to doses used in RCT regarding the primary endpoint of all-cause mortality at three years, on the risk of a composite endpoint (recurrence of ventricular tachyarrhythmias, appropriate ICD therapies) and cardiac rehospitalization.

## Methods

### Study patients, design, and data collection

The present study included retrospectively all patients surviving at least one episode of ventricular tachyarrhythmias from 2002 until 2015 at one institution. All relevant clinical data related to the index event was documented using patients’ files, daily records, documentation from diagnostic examinations and laboratory values, electrocardiograms (ECG), device recordings, and all further information derived from the electronic hospital information system.

Ventricular tachyarrhythmias comprised VT and VF, as defined by current international guidelines [21]. Sustained VT was defined by VT with a duration of more than 30 s or additional hemodynamic collapse within 30 s. Non-sustained VT are defined by less than 30 s. VT comprised wide QRS complex (≥ 120 ms) at a rate greater than 100 beats/minute.^21^ Ventricular tachyarrhythmia was documented by 12-lead ECG, ECG tele-monitoring, ICD or in case of unstable course or during resuscitation by external defibrillator monitoring. Documented VF was treated by external defibrillation and in case of prolonged instability with additional intravenous anti-arrhythmic drugs during CPR. Further documented data contained baseline characteristics, prior medical history, prior medical treatment, length of index stay, detailed findings of laboratory values at baseline, data derived from all non-invasive or invasive cardiac diagnostics, and device therapies. These included coronary angiography, electrophysiological examination, prior or newly implanted ICDs, pacemakers, or cardiac contractility modulators (CCM), which were already implanted at index or at follow-up. Imaging modalities comprised echocardiography or cardiac magnetic resonance imaging (cMRI). The overall presence of an activated ICD summarizes the total sum of all patients with either a prior implanted ICD before admission, those undergoing new ICD implantation at index stay, as well as those with ICD implantation at the complete follow-up period after index hospitalization, referring to sole ICD, subcutaneous-ICD (s-ICD), and cardiac resynchronization therapy with defibrillator function (CRT-D). Documentation period lasted from index event until 2016. Documentation of all medical data was performed by independent cardiologists at the patients’ individual period of hospitalization blinded to final data analyses.

The present study is derived from an analysis of the “Registry of Malignant Arrhythmias and Sudden Cardiac Death—Influence of Diagnostics and Interventions (RACE-IT)” and represents a single-center registry including consecutive patients presenting with ventricular tachyarrhythmias and aborted cardiac arrest being acutely admitted to the University Medical Center Mannheim (UMM), Germany (clinicaltrials.gov identifier: NCT02982473) from 2002 until 2016. The registry was carried out according to the principles of the Declaration of Helsinki and was approved by the medical ethics committee II of the Medical Faculty Mannheim, University of Heidelberg, Germany.

The medical center covers a general emergency department (ED) for emergency admission of traumatic, surgical, neurological, and cardiovascular conditions. Interdisciplinary consultation is an inbuilt feature of this 24/7 service, and connects to a stroke unit, four intensive care units (ICU) with extracorporeal life support, and a chest pain unit (CPU) to alleviate rapid triage of patients. The cardiologic department itself includes a 24 h catheterization laboratory, an electrophysiologic laboratory, a hybrid operating room, and telemetry units.

### Inclusion and exclusion criteria

Consecutive patients with BB therapy were included. Decision to treat patients with BB was based on the discretion of the cardiologists during routine care according to European guidelines [4, 11–13]. Risk stratification was performed according to daily BB dose at index hospital discharge. BB doses were grouped as > 0–12.5%, > 12.5–25%, > 25–50%, and > 50% according to doses used in RCT as follows: target doses for the most commonly used beta-blockers were as follows: metoprolol 200 mg/day [14, 15]; carvedilol 50 mg/day [16]; propranolol: 180 mg/day [17]; bisoprolol 10 mg/day [2]; nebivolol: 10 mg/day [9]. Due to the multi-pharmacological effect, patients with sotalol treatment were excluded from this study. No other BB therapies were included in the study. Patients without BB, with no evidence of daily BB dose, and patients with death during index hospitalization were excluded from the present study. All other medical therapies apart from BB were allowed.

### Primary and secondary endpoints

Follow-up period was set at three years for all outcomes. The primary endpoint was all-cause mortality. All-cause mortality was documented using our electronic hospital information system and by directly contacting state resident registration offices (“bureau of mortality statistics”) all across Germany. Identification of patients was verified by place of name, surname, day of birth, and registered living addresses. Secondary endpoints were a composite endpoint (i.e., recurrences of ventricular tachyarrhythmias and appropriate ICD therapies) and cardiac rehospitalization. Cardiac rehospitalization comprised of rehospitalization due to VT, VF, AMI, acute heart failure, and inappropriate device therapy.

### Statistical methods

Quantitative data is presented as mean ± standard error of mean (SEM), median and interquartile range (IQR), and ranges depending on the distribution of the data, and were compared using Student’s *t* test for normally distributed data or the Mann–Whitney *U* test for nonparametric data. Deviations from a Gaussian distribution were tested by the Kolmogorov–Smirnov test. Spearman’s rank correlation for nonparametric data was used to test univariate correlations. Qualitative data are presented as absolute and relative frequencies, and compared using the Chi^2^ test or the Fisher’s exact test, as appropriate.

First, univariable Kaplan–Meier method was applied to evaluate prognostic differences within the entire cohort. Then, the impact of > 0–12.5%, > 12.5–25%, > 25–50%, and > 50% of BB target dose was analyzed separated for patients with AMI and no AMI, as LVEF ≥ 35% and < 35%, as well as patients with ischemic and non-ischemic cardiomyopathy. Second, multivariable Cox regression models were developed using the “forward selection” option, where only statistically significant variables (*p* < 0.05) were included and analyzed simultaneously. Predefined variables being used for multivariable Cox regressions included: baseline parameters (age, male gender), type of index ventricular tachyarrhythmia, AMI, cardiogenic shock, coronary artery disease, cardiopulmonary resuscitation, presence of an ICD and > 0–12.5% (reference group), > 12.5–25%, > 25–50%, and > 50% of BB target dose.

The result of a statistical test was considered significant for *p* < 0.05. SAS, release 9.4 (SAS Institute Inc., Cary, NC, USA) and SPSS (Version 25, IBM, Armonk, New York) were used for statistics.

## Results

### Study population

**Fig. 1:**
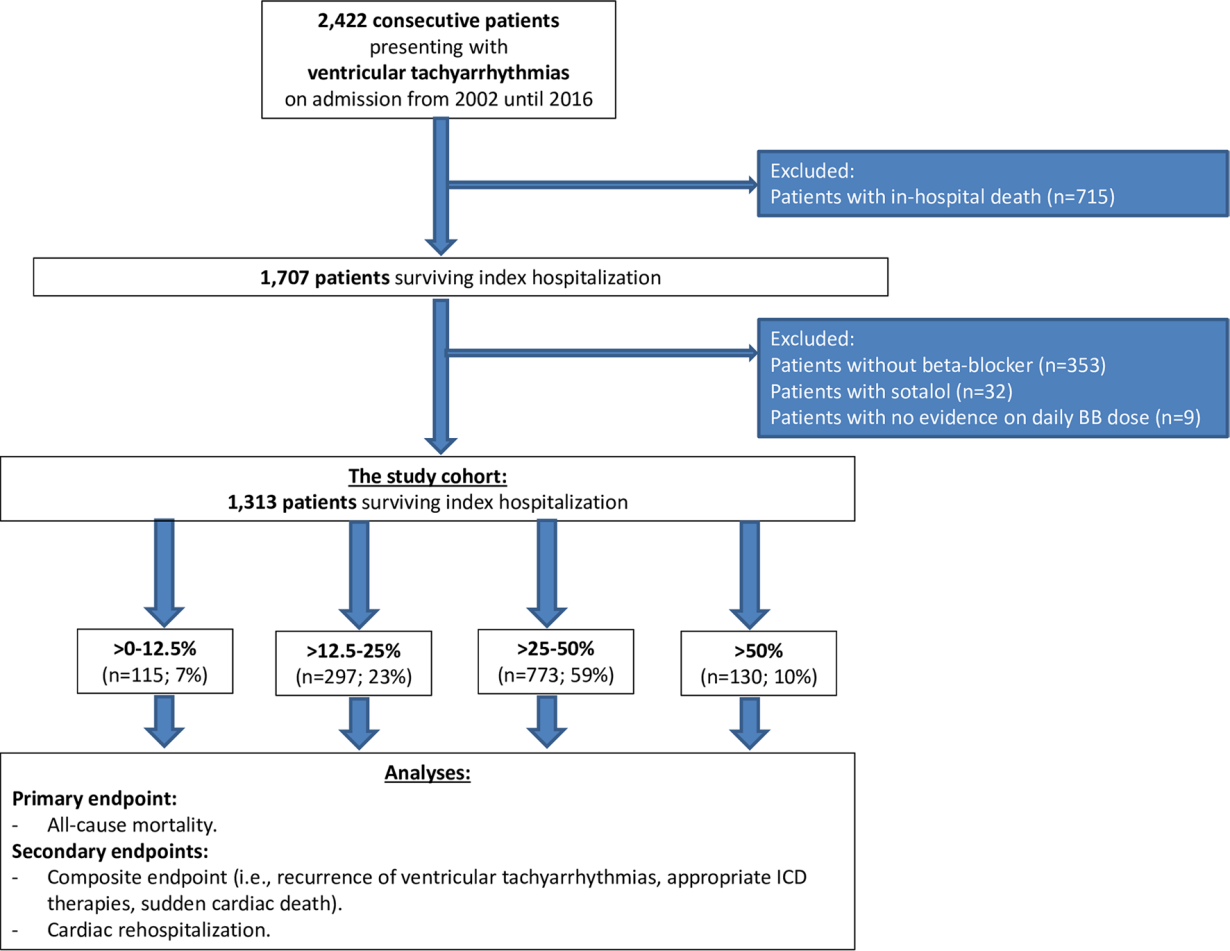
Flow chart of the study population

From a total of 2422 patients with ventricular tachyarrhythmias, 715 were excluded due to in-hospital death, 353 without BB treatment, 32 patients with sotalol, and 9 patients with no evidence of BB dose at hospital discharge (Fig. 1; flow chart). The final study cohort comprised of 1313 patients surviving index episodes of ventricular tachyarrhythmias being discharged on BB therapy. Most patients were discharged with > 25–50% of recommended BB target dose (59%), followed by > 12.5–25% (23%), > 50% (10%), whereas only 7% were discharged with > 0–12.5% of BB target dose.

As seen in Table 1, patients were median-aged at 67 years and most patients were males (73–79%). Distribution of index ventricular tachyarrhythmias was comparable across all subgroups (VT: 65–75% vs. VF: 25–35%; *p* = 0.132). Baseline heart rate did not differ among patients with different BB doses. Especially, the rates of prior myocardial infarction and coronary artery disease were equally distributed (*p* ≤ 0.086). In contrast, LVEF < 35% was most common in patients with > 50% of BB target dose (52% vs. 26–45%; *p* = 0.001). Accordingly, highest ICD implantation rates were seen in the > 50% group (69% vs. 44–45%; *p* = 0.001). Moreover, concomitant treatment with angiotensin receptor blockers (ARB), aldosterone antagonists, and amiodarone was most common in patients on > 50% of recommended BB target dose (*p* ≤ 0.013) (Table 1).

**Table 1:** Baseline characteristics

Characteristic	> 0–12.5% (*n* = 113; 7%)		> 12.5–25% (*n* = 297; 23%)		> 25–50% (*n* = 773; 59%)		> 50% (*n* = 130; 10%)		*p* value
Age, median (range)	70 (32–87)		68 (45–80)		66 (22–87)		68 (32–82)		**0.010**
Male gender, *n* (%)	85	(75)	224	(75)	566	(73)	103	(79)	0.501
Baseline heart rate, median (IQR)	71	(61–88)	68	(60–82)	70	(60–81)	73	(65–84)	0.584
Ventricular tachyarrhythmia at index, *n* (%)									
Ventricular tachycardia	85	(75)	192	(65)	512	(66)	93	(72)	0.132
Ventricular fibrillation	28	(25)	105	(35)	261	(34)	37	(29)	
Cardiovascular risk factors, *n* (%)									
Arterial hypertension	65	(58)	159	(54)	506	(66)	81	(62)	**0.003**
Diabetes mellitus	30	(27)	63	(21)	221	(29)	28	(22)	0.055
Hyperlipidemia	35	(31)	78	(26)	278	(36)	49	(38)	**0.015**
Smoking	37	(33)	104	(35)	241	(31)	36	(28)	0.456
Cardiac family history	4	(4)	37	(13)	97	(13)	16	(12)	**0.046**
Comorbidities at index stay, n (%)									
Prior myocardial infarction	27	(24)	75	(25)	215	(28)	43	(33)	0.313
Prior coronary artery disease	51	(45)	117	(39)	351	(45)	68	(52)	0.086
Prior heart failure	39	(35)	58	(20)	214	(28)	45	(34)	**0.001**
Atrial fibrillation	37	(33)	78	(26)	235	(30)	54	(42)	**0.017**
Paroxysmal	29	(26)	57	(19)	161	(21)	30	(23)	
Persistent	1	(0.9)	9	(3)	22	(3)	9	(7)	**0.013**
Permanent	7	(6)	12	(4)	52	(7)	15	(12)	
Non-ischemic cardiomyopathy	12	(11)	13	(4)	63	(8)	18	(14)	**0.006**
Arrhythmogenic right ventricular dysplasia	0	(0)	4	(1)	4	(0.5)	1	(0.8)	0.388
Hypertrophic obstructive cardiomyopathy	0	(0)	2	(0.7)	2	(0.3)	2	(2)	0.184
Hypertrophic non-obstructive cardiomyopathy	0	(0)	1	(0.3)	8	(1)	3	(2)	0.168
Dilated cardiomyopathy	12	(11)	6	(2)	49	(6)	12	(9)	0.188
Cardiopulmonary resuscitation	26	(23)	107	(36)	249	(32)	27	(21)	**0.027**
In hospitall	11	(10)	37	(13)	87	(11)	9	(7)	
Out of hospital	15	(13)	70	(24)	162	(21)	18	(14)	
Chronic kidney disease	49	(44)	102	(35)	330	(43)	58	(45)	0.061
COPD	10	(9)	13	(4)	62	(8)	11	(9)	0.172
Stroke	13	(4)	2	(2)	15	(3)	3	(2)	0.715
Intracranial hemorrhage	4	(1)	0	(0)	1	(0)	2	(2)	**0.043**
Coronary angiography, *n* (%)	78	(69)	216	(73)	555	(72)	71	(55)	**0.001**
No evidence of CAD	21	(27)	53	(25)	135	(24)	23	(32)	0.558
1-vessel disease	24	(31)	51	(24)	131	(24)	18	(25)	
2-vessel disease	18	(23)	46	(21)	136	(25)	13	(18)	
3-vessel disease	15	(19)	66	(31)	153	(28)	17	(24)	
Chronic total occlusion	13	(17)	35	(16)	115	(21)	16	(23)	0.417
Presence of CABG	11	(14)	29	(13)	85	(15)	12	(17)	0.871
PCI	27	(35)	105	(49)	241	(43)	24	(34)	0.058
Acute myocardial infarction	26	(23)	99	(33)	230	(30)	20	(15)	**0.001**
STEMI	6	(5)	42	(14)	88	(11)	6	(5)	**0.006**
NSTEMI	20	(18)	57	(19)	142	(18)	14	(11)	0.174
Hyperkalemia	3	(1)	1	(1)	2	(0)	0	(0)	0.478
Hypokalemia	23	(7)	6	(6)	29	(6)	10	(8)	0.370
Short QT syndrome	1	(0.9)	0	(0)	0	(0)	0	(0)	1.000
Long QT syndrome	3	(3)	8	(3)	15	(2)	4	(3)	0.785
Brugada syndrome	1	(0.9)	0	(0)	0	(0)	0	(0)	1.000
LVEF, *n* (%)									
≥ 55%	23	(24)	92	(36)	173	(26)	20	(19)	**0.001**
54–45%	10	(11)	47	(19)	105	(16)	14	(13)	
44–35%	19	(20)	49	(19)	143	(21)	18	(17)	
< 35%	43	(45)	66	(26)	247	(37)	56	(52)	
Not documented	18	–	43	–	205	–	22	–	–
Cardiac therapies at index, *n* (%)									
Electrophysiological examination	48	(43)	87	(29)	235	(30)	53	(41)	**0.007**
VT ablation therapy	6	(5)	14	(5)	52	(7)	18	(14)	**0.006**
Presence of an ICD, *n* (%)	62	(55)	130	(44)	411	(53)	89	(69)	**0.001**
Medication at discharge, *n* (%)									
ACE-inhibitor	81	(72)	208	(70)	536	(69)	83	(64)	0.534
ARB	10	(9)	25	(9)	85	(11)	27	(21)	**0.002**
Statin	66	(58)	203	(68)	533	(69)	85	(65)	0.141
Amiodarone	23	(20)	33	(11)	124	(16)	29	(22)	**0.013**
Digitalis	20	(18)	34	(11)	102	(13)	17	(13)	0.424
Aldosterone antagonist	14	(12)	26	(9)	87	(11)	31	(24)	**0.001**
Vitamin K antagonist	2	(2)	3	(1)	22	(3)	6	(5)	0.128
Direct oral anticoagulant	25	(22)	50	(17)	157	(20)	40	(31)	**0.012**
Aspirin only	33	(29)	71	(24)	210	(28)	36	(27)	0.634
Dual antiplatelet therapy	30	(27)	120	(40)	253	(33)	31	(24)	**0.002**

*ACE* angiotensin-converting enzyme, *ARB* angiotensin receptor blocker, *CABG* coronary artery bypass grafting, *CAD* coronary artery disease, *COPD* chronic obstructive pulmonary disease, *LVEF* left-ventricular ejection fraction, *NSTEMI* non-ST-segment myocardial infarction, *PCI* percutaneous coronary intervention, *SEM* standard error of mean, *STEMI* ST-segment MI, *VT* ventricular tachycardia

Bold type indicates *p* < 0.05

### Follow-up data, primary and secondary endpoints within the entire study cohort

**Fig. 2:**
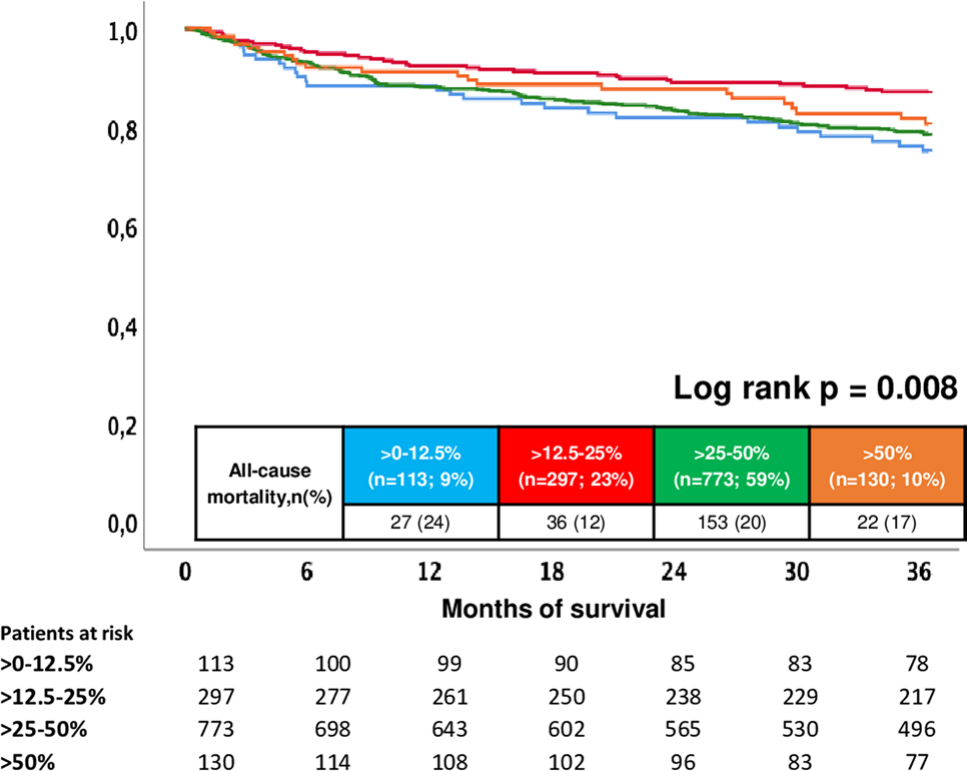
Prognostic impact of different BB doses on all-cause mortality

**Fig. 3:**
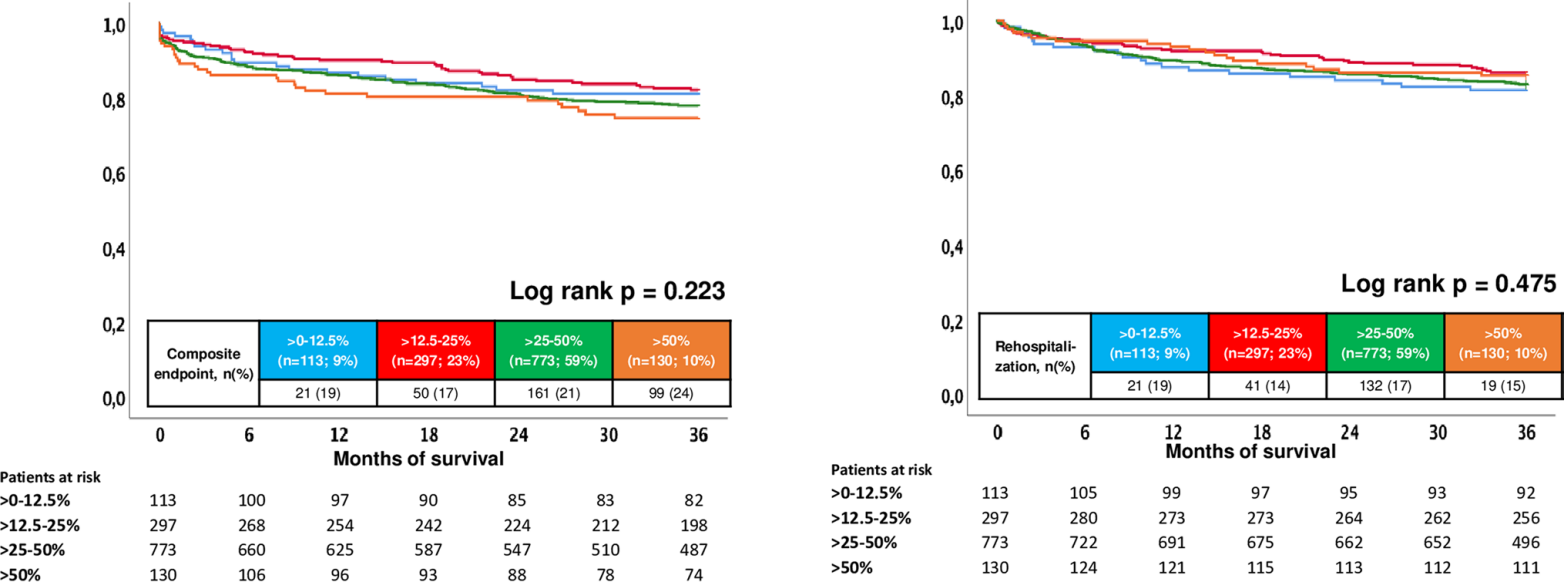
Prognostic impact of different BB doses on the composite endpoint (i.e., recurrence of ventricular tachyarrhythmias, sudden cardiac death) (left) and cardiac rehospitalization (right)

Median follow-up time within the entire study cohort was 4.8 years (IQR 2.3–8.3 years). At three years of follow-up, the primary endpoint all-cause mortality occurred in 12% of patients with > 12.5–25%, 17% with > 50%, 20% with > 25–50% and in 24% with > 0–12.5% of BB target dose. Accordingly, risk of all-cause mortality was improved in patients with > 12.5–25% of recommended BB target dose as compared to those with > 0–12.5% (HR = 0.489; 95% CI 0.297–0.806; *p* = 0.005) (Table 2 and Fig. 2). In contrast, > 25–50% (HR = 0.849; 95% CI 0.564–1.279; *p* = 0.434) and > 50% of BB dose (HR = 0.735; 95% CI 0.419; 95% CI 0.419–1.291; *p* = 0.285) did not improve all-cause mortality compared to patients on > 0–12.5% of BB target dose. Regarding secondary endpoints, risk of the composite endpoint (i.e., recurrence of ventricular tachyarrhythmias, SCD) was not affected by BB target dose (> 12.5–25%: HR = 0.909; 95% CI 0.546–1.514; *p* = 0.715; > 25–50%: HR = 1.175, 95% CI 0.745–1.815; *p* = 0.488 and > 50%: 1.398; 95% CI 0.803–2.432; *p* = 0.236; > 0–12.5%: reference group) (Table 2 and Fig. 3, left panel). In line, risk of cardiac rehospitalization was comparable among all subgroups (> 12.5–25%: HR = 0.719; 95% CI 0.425–1.216; *p* = 0.218; > 25–50%: HR = 0.908, 95% CI 0.573–1.438; *p* = 0.680 and > 50%: 0.765; 95% CI 0.412–1.424; *p* = 0.399; > 0–12.5%: reference group) (Table 2 and Fig. 3, right panel).

**Table 2:** Primary and secondary endpoints, and follow-up data

Characteristic	> 0–12.5% (*n* = 113; 7%)		> 12.5–25% (*n* = 297; 23%)		> 25–50% (*n* = 773; 59%)		> 50% (*n* = 130; 10%)		*p* value
Primary endpoint, *n* (%)									
All-cause mortality, at 3 years	27 (24)		36 (12)		153 (20)		22 (17)		**0.010**
Secondary endpoints, *n* (%)									
Rehospitalization, at 3 years	21 (19)		41 (14)		132 (17)		19 (15)		0.493
Composite endpoint (recurrent ventricular tachyarrhythmias; appropriate ICD therapies), at 3 years	21 (19)		50 (17)		161 (21)		31 (24)		0.317
Follow-up times, *n* (%)									
Hospitalization time; days (median (IQR))	14 (8–29)		12 (8–21)		13 (8–23)		11 (7–17)		**0.001**
ICU time; days (median (IQR))	3 (0–7)		3 (0–7)		3 (0–8)		2 (0–6)		**0.001**
Survival time; days (median (IQR))	1687 (767–2748)		2050 (991–3284)		1625 (652–2855)		1381 (678–2631)		**0.001**

*ICU* invasive care unit, *IQR* interquartile range

Level of significance *p* ≤ 0.05

Bold type indicates *p* ≤ 0.05

### Stratification by AMI, LVEF, and type of cardiomyopathy

**Fig. 4:**
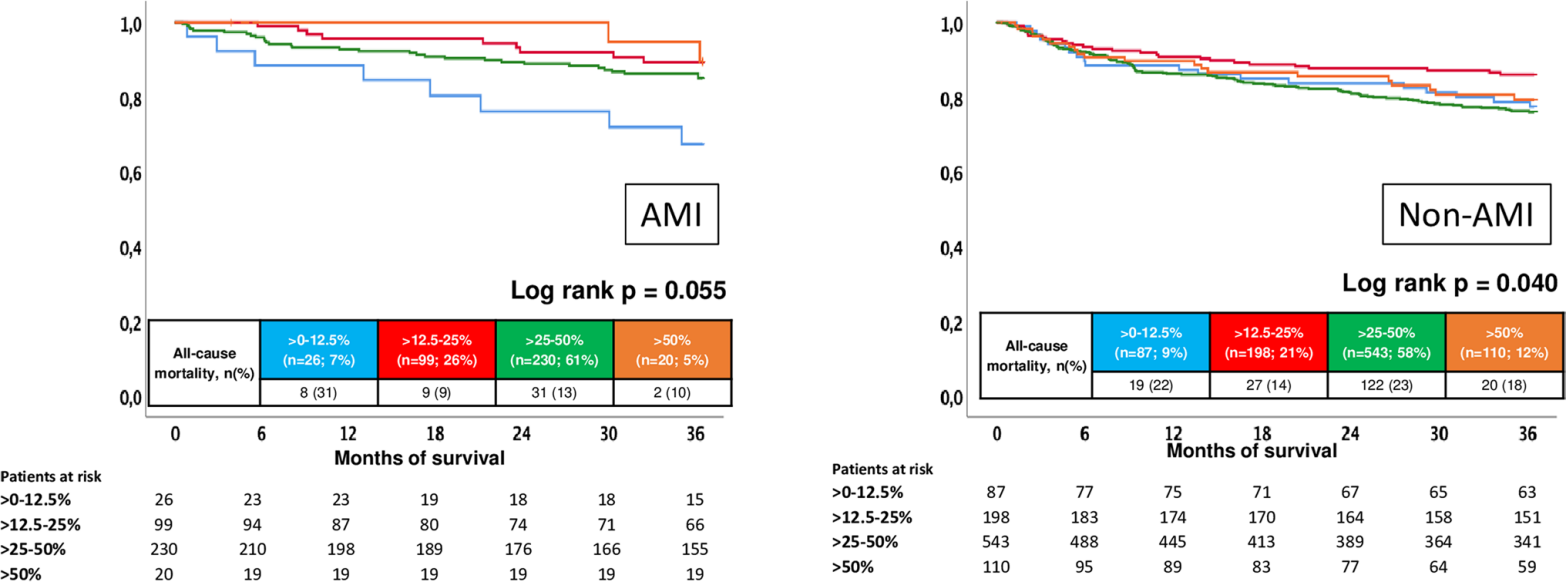
Prognostic impact of different BB doses on all-cause mortality in patients with AMI (left) and without AMI (right)

Subsequently, prognosis of BB dose was investigated in the subgroups of AMI and non-AMI patients and stratified by LVEF. In patients with AMI, > 12.5–25%, > 25–50%, and > 50% of BB target dose were not associated with improved survival compared to patients on > 0–12.5% of BB dose (log rank *p* = 0.055) (Fig. 4, left panel). In patients without AMI, only the > 12.5–25% group was associated with improved survival at three years (HR = 0.605; 95% CI 0.336–1.088; *p* = 0.093; statistical trend) compared to patients discharged on > 0–12.5% of BB target dose (Fig. 4, right panel).

**Fig. 5:**
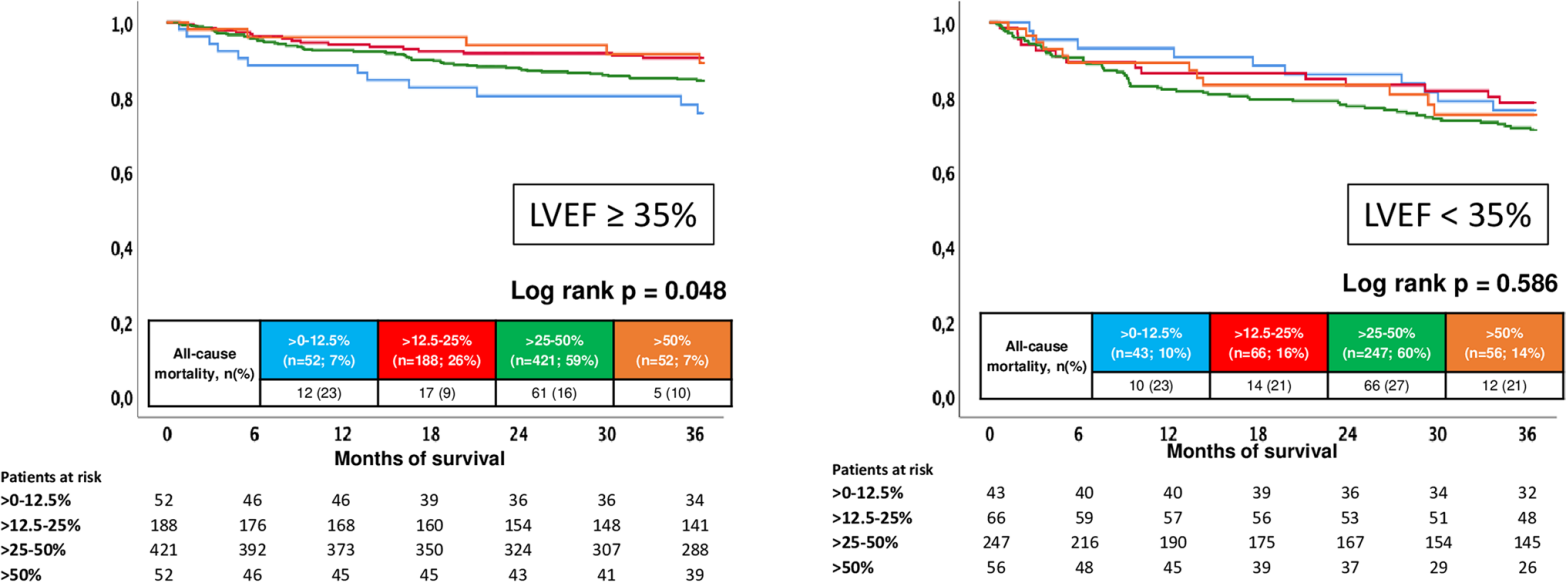
Prognostic impact of different BB doses on all-cause mortality in patients with LVEF ≥ 35% (left) and LVEF < 35% (right)

Focusing on patients with LVEF ≥ 35%, improved survival was observed in patients on > 12.5–25% of BB target dose compared to > 0–12.5% (HR = 0.364; 95% CI 0.174–0.762; *p* = 0.007), whereas prognosis was not improved in patients with > 25–50% (HR = 0.599; 95% CI 0.322–1.111; *p* = 0.104) and > 50% of recommended BB target dose (HR = 0.395; 95% CI 0.139–1.122; *p* = 0.081) (Fig. 5, left panel). In patients with LVEF < 35%, no differences regarding long-term prognosis were observed in patients treated with different BB doses (log rank *p* = 0.586) (Fig. 5, right panel).

**Fig. 6:**
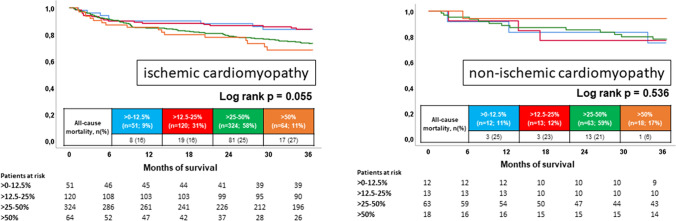
Prognostic impact of different BB doses on all-cause mortality in patients with ischemic (left) and non-ischemic cardiomyopathy (right)

However, BB dose did not affect long-term mortality in patients with ischemic (log rank *p* = 0.055) and non-ischemic cardiomyopathy (log rank *p* = 0.563) (Fig. 6).

### Multivariable Cox regression models

After multivariable adjustment, only > 12.5–25% of BB target dose was associated with improved all-cause mortality at three years (HR = 0.594; 95% CI 0.359–0.981; *p* = 0.042). In contrast, > 25–50% (HR = 0.938; *p* = 0.759) and 50% (HR = 0.830; *p* = 0.530) were not associated with mortality (Table 3). Besides BB target dose, especially increasing age (HR = 1.636; *p* = 0.001), cardiogenic shock (HR = 1.695; *p* = 0.001), and cardiopulmonary resuscitation (HR = 1.319; *p* = 0.025) were associated with increased risk of all-cause death, whereas the presence of AMI (HR = 0.563; *p* = 0.002) and an ICD (HR = 0.644; *p* = 0.001) was associated with favorable long-term outcomes.

**Table 3:** Multivariable Cox regression analyses

Endpoint	HR	95% CI	*p* value
Mortality			
Age (decade)	1.636	1.429–1.871	**0.001**
Gender	1.309	0.955–1.795	0.095
Ventricular tachycardia	0.749	0.508–1.104	0.144
Cardiogenic shock	1.695	1.117–2.571	**0.013**
Acute myocardial infarction	0.563	0.391–0.809	**0.002**
Coronary artery disease	1.133	0.831–1.546	0.429
Presence of ICD	0.644	0.490–0.845	**0.001**
Cardiopulmonary resuscitation	1.319	1.035–1.681	**0.025**
> 12.5–25% of BB target dose	0.594	0.359–0.981	**0.042**
> 25–50% of BB target dose	0.938	0.621–1.415	0.759
> 50% of BB target dose	0.830	0.472–1.462	0.519
> 0–12.5% of BB target dose	(reference group)		
Composite endpoint			
Age (decade)	1.121	1.008–1.247	**0.036**
Gender	0.960	0.712–1.295	0.789
Ventricular tachycardia	0.928	0.647–1.332	0.687
Cardiogenic shock	1.379	0.860–2.212	0.182
Acute myocardial infarction	1.103	0.697–1.472	0.946
Coronary artery disease	0.818	0.619–1.082	0.160
Presence of ICD	7.406	4.993–10.985	**0.001**
Cardiopulmonary resuscitation	0.765	0.595–1.146	0.079
> 12.5–25% of BB target dose	1.195	0.713–2.003	0.499
> 25–50% of BB target dose	1.299	0.822–2.052	0.263
> 50% of BB target dose	1.218	0.698–2.128	0.488
> 0–12.5% of BB target dose	(reference group)		
Rehospitalization			
Age (decade)	1.061	0.944–1.193	0.320
Gender	0.936	0.670–1.306	0.696
Ventricular tachycardia	0.730	0.481–1.107	0.138
Cardiogenic shock	1.598	1.005–2.540	**0.047**
Acute myocardial infarction	1.223	0.847–1.765	0.283
Coronary artery disease	1.261	0.897–1.774	0.181
Presence of ICD	4.196	2.937–5.995	**0.001**
Cardiopulmonary resuscitation	0.971	0.715–1.319	0.850
> 12.5–25% of BB target dose	0.893	0.522–1.527	0.679
> 25–50% of BB target dose	0.984	0.617–1.569	0.946
> 50% of BB target dose	0.726	0.388–1.360	0.318
> 0–12.5% of BB target dose	(reference group)		

*BB* beta-blocker, *CI* confidence interval, *HR* hazard ratio, *ICD* implantable cardioverter defibrillator

Level of significance *p* ≤ 0.05

Bold type indicates statistical significance

No differences were observed for BB dose regarding secondary endpoints, such as the composite endpoint (i.e., recurrence of ventricular tachyarrhythmias, appropriate ICD therapies, SCD) and cardiac rehospitalization after multivariable adjustment (Table 3).

## Discussion

The present study evaluates the prognostic impact of beta-blocker dose on the primary endpoint of all-cause mortality, as well as on secondary endpoints, such as a composite arrhythmic endpoint (i.e., recurrence of ventricular tachyarrhythmias, appropriate ICD therapies) and cardiac rehospitalization at three years in patients surviving an index episodes of ventricular tachyarrhythmias. The present study suggests best long-term survival in patients treated with > 12.5–25% of recommended beta-blocker target dose, which was still evident after multivariable adjustment. Best survival in the presence of > 12.5–25% of BB dose was especially seen in the subgroups of patients with LVEF ≥ 35%. In contrast, higher beta-blocker doses were not associated with improved all-cause mortality. Finally, the risk of the composite endpoint and cardiac rehospitalization were not affected by beta-blocker dose.

Most landmark studies in the field of BB therapy that led to the class Ia indication for BB therapy for primary prevention of SCD enrolled patients with systolic HF and/or AMI within the last century [1, 2, 16]. By now, characteristics of patients have changed significantly due to improved treatment options for underlying cardiac diseases, including improved revascularization strategies, novel pharmacotherapies, better guideline adherence, and increasing supply with ICD and cardiac resynchronization therapy [18]. One may therefore question whether the prognostic impact of BB therapy is the same in the modern era. Since no RCT reevaluated the prognostic value of BB therapy nowadays, current European guidelines demand the need of registry data to reassess the impact of pharmacotherapies for primary prevention of SCD [4]. However, the recommended daily doses of BB treatment rely on those doses used in the initial RCT, but furthermore, no RCT investigated the prognostic value of different BB doses yet. The prognosis of patients treated with different BB doses was, however, investigated within various registries with inconsistent findings [8, 19, 20].

The COMET trial investigated prognostic impact of different doses of metoprolol and carvedilol in 2599 patients with HF and LVEF ≤ 35%, demonstrating achievement of BB target dose at 4 months to be associated with decreased risk of death, whereas long-term prognosis was not assessed [20]. In contrast, a meta-analysis by McAlister et al*.* found that BB dose is not associated with survival including 23 BB trials with patients with systolic HF [8]. Furthermore, a sub-study of the HF-ACTION trial investigated the prognostic role of BB dose in 2,331 ambulatory patients with systolic HF and LVEF < 35%. During a median follow-up of 2.5 years, increased risk of all-cause death or hospitalization in patients with low BB dose (i.e., < 50 mg carvedilol equivalent) was observed, whereas higher BB doses did not improve outcomes. In contrast, risk of arrhythmic events was not affected by BB use [19]. The present study did not find improved outcomes due to BB dose in the presence of LVEF < 35%, but confirmed the findings that arrhythmic events were not affected by BB dose. Furthermore, the present study widens the evidence of BB therapy in patients with ventricular tachyarrhythmias. Most patients with ventricular tachyarrhythmias do not have evidence of systolic HF with LVEF < 35% [4]. In contrast, studies investigating the prognostic role of BB dose usually focus on patients with severely depressed LVEF (i.e., 95% of patients in the meta-analysis by McAlister et al*.*). However, due to the retrospective study design, one may not exclude higher BB doses in patients with more advanced stages of HF (such as more symptomatic HF). Thus, increased New York Heart Association class was recently shown to be an independent predictor of mortality in HF patients [21]. Therefore, further studies or even RCT will be necessary to confirm our hypothesis-generating findings regarding BB treatment and appropriate BB dose for secondary prevention of SCD.

In conclusion, the present study demonstrated improved long-term survival in patients treated with > 12.5–25% of recommended beta-blocker target dose, whereas increasing BB doses were not associated with improved long-term outcomes in patients surviving index episodes of ventricular tachyarrhythmias. In contrast, the risk of the composite endpoint and cardiac rehospitalization were not affected by beta-blocker dose.

### Study limitations

This observational and retrospective registry-based analysis reflects a realistic picture of consecutive health-care supply of high-risk patients presenting with ventricular tachyarrhythmias. Lost to follow-up rate regarding the evaluated endpoint of all-cause mortality was minimal. Pharmacological therapies, as well as their doses were based on discharge medication at index event and were not reassessed during follow-up. Systolic blood pressure was available for minor part of the study cohort only and was therefore beyond the scope of the present study. Furthermore, the proportion of patients undergoing VT ablation therapy was rather small within the present registry. All clinical data was documented reliably by individual cardiologists during routine clinical care being blinded to final analyses, alleviating the use of an independent clinical event committee. Furthermore, cardiac rehospitalization was assessed at our institution only.
